# The PTAP sequence duplication in HIV-1 subtype C Gag p6 in drug-naive subjects of India and South Africa

**DOI:** 10.1186/s12879-017-2184-4

**Published:** 2017-01-24

**Authors:** Shilpee Sharma, Shambhu G. Aralaguppe, Melissa-Rose Abrahams, Carolyn Williamson, Clive Gray, Pachamuthu Balakrishnan, Shanmugam Saravanan, Kailapuri G. Murugavel, Suniti Solomon, Udaykumar Ranga

**Affiliations:** 1 Jawaharlal Nehru Centre for Advanced Scientific Research, HIV-AIDS Laboratory, Jakkur (PO), Bangalore, 560 064 India; 2 Division of Medical Virology and Division of Immunology, Institute of Infectious Disease and Molecular Medicine, Department of Pathology, University of Cape Town, and National Health Laboratory Service, Cape Town, South Africa; 3 YRG Centre for AIDS Research and Education, Chennai, India

**Keywords:** HIV-1, Subtype C, Gag, p6, PTAP duplication, HIV evolution

## Abstract

**Background:**

HIV-1 subtype C demonstrates several biological properties distinct from other viral subtypes. One such variation is the duplication of PTAP motif in p6 Gag. PTAP motif is a key player in viral budding. Here, we studied the prevalence of PTAP motif duplication in subtype C viral strains in a longitudinal study.

**Methods:**

In a prospective follow-up study, 65 HIV-1 seropositive drug-naive subjects were monitored in two different clinical cohorts of India for 2 years with repeated sampling at 6-month intervals. The viral RNA was extracted from plasma, the *gag* segment was amplified and sequenced. From a subset of viral isolates the sequences of *pol*, *env* and *LTR* were sequenced. Using HIV-1 *gag* amino acid sequences available from public databases and additional sequences derived from the Indian and South-African cohorts, we examined the nature of PTAP motif duplication in subtype C.

**Results:**

In 16% (8 of 50) of the primary viral strains of India, we identified a sequence duplication of the PTAP motif in Gag p6. The length of the sequence duplication varied from 6 to 14 amino acids in the viral isolates but remained fixed within a subject over a period of 24–36 month follow-up. In the duplicated motif, the core PTAP motif was invariable, but the flanking residues were highly variable. In an acute phase clinical cohort of South Africa, in a subset of 75 subjects, we found the presence of the PTAP duplication at a frequency of 29.3%. An analysis of the *gag* sequences from the extant databases showed that unlike other subtypes of HIV-1, subtype C has a natural propensity to generate the PTAP motif duplication at a significantly higher frequency and of greater length. Additionally, the global prevalence of PTAP duplication in subtype C appears to be increasing progressively over the past 30 years.

**Conclusion:**

We showed that in subtype C, the duplication of the PTAP motif in p6 Gag involves sequence stretches of greater length, and at a much higher frequency as compared to other HIV-1 subtypes. Given that subtype C naturally lacks the Alix binding motif, the acquisition of an additional PTAP motif may confer replication advantage on this HIV-1 subtype. Further investigation is warranted to examine the significance of PTAP motif duplication on the replicative fitness of HIV-1.

## Background

HIV-1 subtype C is responsible for approximately half of all global infections [1]. HIV-1 subtypes differ from each other in genetic sequence, geographical distribution, co-receptor usage, pathogenic potential, and replication and transmission properties [2, 3]. It is increasingly appreciated that the subtype-unique genetic variations may have a significant impact on the viral biological properties that in turn may influence the differences in the prevalence of the viral subtypes and pathogenesis.

HIV-1 subtype C appears to have a unique potential to introduce sequence duplications at specific locations in the viral genome at a much higher frequency than the other HIV-1 subtypes. Importantly, the sequence insertions in subtype C are significantly longer permitting the acquisition of additional copies of biologically functional motifs, by the duplication of the adjacent sequences. This phenomenon is expected to enhance the replication fitness of the variant viral strains. For example, the duplication of a 21 bp sequence motif in the viral promoter was shown to create a fourth NF-κB motif (typically, the canonical subtype C promoter contains three NF-κB binding sites) in the Long terminal repeat (LTR) [4]. We recently demonstrated that the 4-κB viral strains have been spreading in the population at a rapid rate in India dominating the 3-κB viruses [5].

**Fig. 1:**
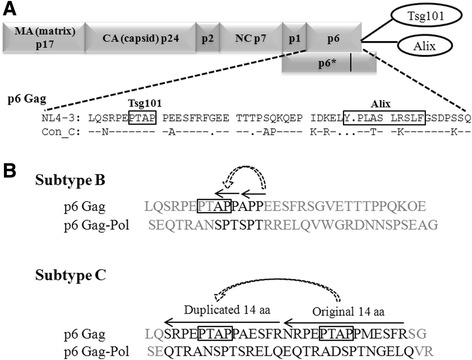
**a** Schematic representation of HIV-1 Gag protein domains. The four major domains of Gag (MA, CA, p7, and p6) are depicted including the two linker sequences p1 and p2. HIV-1 Gag interacts with the ESCRT complex proteins Tsg101 and Alix to regulate viral budding. The sequence of subtype B NL4-3 *gag p6*is presented and the sequence motifs PTAP and YPXnL, which serve as the binding motif for Tsg101 and Alix, respectively, are highlighted using the square boxes. Con_C represents the subtype C gag p6 consensus amino acid sequence. The dashes represent sequence identity and the dots sequence deletion. **b** A comparative analysis of the PTAP sequence duplication in subtypes B and C. In subtype B, a partial PTAP duplication consisting of three amino-acid residues (APP) is common. In contrast in subtype C, a sequence duplication of 14 amino acids is common. A 14 amino acid sequence duplication of subtype C derived from the primary clinical isolate T004 is presented. The amino acid sequences, the original and the duplicated sequences, in Gag and Gag-Pol are depicted. The arrows represent the length of sequence duplication and the direction of polymerization by the reverse transcriptase while synthesizing the cDNA from the viral RNA. The core PTAP motifs are highlighted using the square boxes. The sequences flanking the 3 or 14 aa residues are shown in gray

The second case of sequence duplication creating an additional functional motif of biological significance is evident in the PTAP motif (the motif consisting of the four Proline, Threonine, Alanine, and Proline amino acids) of Gag p6, a 52 amino-acid protein [6–8]. HIV-1 *gag* varies genetically up to 8–19% among the diverse viral subtypes [9–11] and possesses subtype-specific genetic variations [12, 13]. The p6 domain of Gag is highly flexible and can accommodate a wide range of length variations due to sequence insertion or deletion that possibly modulates Gag functions [14, 15]. Numerous insertions and deletions have been reported in Gag p6 of which the most common insertions are in the PTAP, KQE and FRFG motifs of subtype B [16] and subtype C [17]. The polyfunctional Gag p6 is associated with the recruitment of several host factors required for the viral particle formation. The PTAP motif located in the L-domain of p6 recruits the primary budding factor tumor susceptibility gene 101 (Tsg101) and the other components of the endosomal sorting complex (ESCRT) machinery to the site of virus assembly [18]. Additionally, a second domain, the YPXnL motif, binds ALG-2 interacting protein 1/X (ALIX) [19] which is believed to enhance the viral budding (Fig. 1a). Thus, Gag p6 plays a critical role in viral budding especially via an interaction with the Tsg101 and the ESCRT machinery using the PTAP motif.

Importantly, several studies previously reported the accumulation of sequence insertions at the N-terminal region within the PTAP motif of Gag p6 [8, 20–22]. The sequence duplication at this location may be divided into two categories based on whether or not the core PTAP motif is duplicated. Based on this classification, most of the sequence insertions reported in the primary clinical isolates of subtype B are characterized by only a partial sequence duplication that does not lead to the creation of an additional PTAP motif but only a part of the motif. In subtype B, while a large number of the sequence insertions duplicated a three amino acid stretch ‘APP’ in Gag p6 and ‘SPT’ in Gag-Pol p6 (Fig. 1b), only a small fraction of the sequence insertions constituted a complete duplication of the PTAP motif. Of note, the DNA sequence encoding Gag p6 is also translated in a different reading frame, following a frame-shift, to encode a different protein called Gag-Pol transframe domain p6* or Gag-Pol p6 of 56 amino acids. The sequence variations in this region of the virus, therefore, are likely to influence two different viral protein derivatives Gag p6 and Gag-Pol p6.

The administration of the antiretroviral therapy (ART) appears to have a significant impact on the nature and frequency of the amino acid sequence insertion in the PTAP motif although the significance of this observation to drug resistance is not known [23]. A positive correlation was identified between nucleoside-based ART and PTAP duplication in HIV-1 subtype B infection [7, 24–26]. Furthermore, amino-acid polymorphism in Gag p6 can affect viral replication [27] with an increase in infectivity and resistance to reverse transcriptase (RT) inhibitors [16, 27]. A significant increase in the prevalence of the PTAP motif sequence insertion following ART exposure in subtypes B (*p* = 0.0294) and C (*p* = 0.0001) alludes to a potential role of the duplication in antiretroviral drug-resistance [28]. The proposed association between ART treatment and the PTAP duplication, however, is controversial based on two other observations. First, some studies failed to find a significant difference in the frequency of the PTAP insertion between ART-naïve and ART-exposed arms in subtype B [29]. Second, the high frequency of the PTAP sequence duplication in drug-naive subjects of subtype C alludes to a different role for the sequence insertion other than drug resistance [28].

The biological significance of the PTAP motif sequence duplication, predominantly partial in subtypes B and others (mostly an insertion of three amino acid sequence) and complete in subtype C (sequence insertions of four amino acids or longer, mostly as long as 14 amino acids) has not been evaluated adequately. In this backdrop, a publication from Brazil examined PTAP duplication in a clinical cohort of drug-naïve and drug-experienced arms representing HIV-1 infection of three different subtypes B, C and F [28]. This study identified a significant elevation in the frequency of PTAP duplication in subtype C as compared to the other two subtypes in both the arms of the study. In the backdrop of the paucity of studies examining the higher and importantly, longer sequence duplication of the Gag p6 PTAP motif in subtype C, the primary objective of the present work is to evaluate the prevalence of the PTAP duplication in the primary infections of HIV-1 subtype C in India. In a longitudinal follow-up of 2 to 3 years (2010–2013) of two different cohorts of southern India consisting of 65 HIV-1 seropositive subjects, we ascertain the higher frequency of the PTAP motif duplication in subtype C. Additionally and importantly, we demonstrate that the length of the PTAP duplication in subtype C is significantly longer as compared to subtype B.

## Methods

### Ethics statement

Ethical approval for the present study was granted by the Institutional Review Board of YRG CARE (Y. R. Gaitonde Centre for AIDS Research and Education), Chennai. A written informed consent was obtained from all the subjects enrolled in the study and maintained with confidence. The Human Ethics and Biosafety Committee of JNCASR (Jawaharlal Nehru Centre for Advanced Scientific Research), Bangalore reviewed the proposal and approved the study.

### Characteristics of the study and the clinical samples

From the clinical records available at YRG CARE over the past several years, seropositive subjects were enrolled in two different clinical cohorts, called the YRG CARE (Chennai, Tamil Nadu) and Nellore (Andhra Pradesh) cohorts, for the longitudinal study spanning over a period of 2–3 years. 30 subjects belonged to YRG CARE cohort, Chennai, Tamil Nadu and 35 subjects to the Nellore cohort, Andhra Pradesh. The participants of the YRG CARE cohort were diagnosed between years 1996 and 2008, and the dates of diagnosis of the Nellore cohort are not available. The clinical samples were collected at an interval of 6 months. The primary inclusion criteria consisted that the CD4 counts should be above 500 cells/μl at the time of enrollment and that the subjects should be drug-naive and free of AIDS-related clinical symptoms. The exclusion criteria included the presence of opportunistic infections, signs of acute systemic illness and prior antiretroviral treatment. The study participants consisted of only adult subjects over 18 years of age. The subjects were all drug-naive and believed to have acquired the infection primarily through heterosexual transmission. The clinical profile of the subjects including the subject ID, sampling date, age, gender, viral load and CD4 count has been summarized (Table 1).

**Table 1:** The clinical profile of the study subjects belonging to two different clinical cohorts of India

(A) The YRG CARE cohort, *n* = 30																
Subject ID	Year of Diagnosis	Enrolment date	Age/ Gender	Time (months from the date of enrolment)												GenBank Accn. No.
				PVL			CD4						CD8			
				M0	M12	M24	M0	M6	M12	M18	M24	M30	M0	M12	M24	*gag*
T-001	22–05–2002	26–11–2010	28/F	12074	29617	60953	560	569	525	530	645	355	1633	1804	1273	KT152633
T-002	22–01–2002	06–12–2010	34/F	15354	16293	6852	517	855	740	879	565	584	610	762	734	KP890700–11
T-004	15–03–2004	16–12–2010	29/F	33784	36627	112577	553	442	508	359	321	296	>2000	>2000	1910	KT152674–686
T-005	13–12–2003	20–12–2010	26/F	912	x	x	770	x	x	x	x	x	>2000	x	x	KT152634
T-006	25–08–2008	29–12–2010	28/F	1638	24773	x	527	480	409	547	x	x	1513	1199	x	KP890712–21
T-007	27–10–2007	30–12–2010	26/F	<150	263	722	958	799	1016	886	1311	x	859	992	1380	­
T-008	17–10–2008	20–01–2011	30/F	34640	172183	x	468	480	484	432	476	415	1158	1083	901	KP890722-38
T-009	24–08–2006	24–01–2011	27/F	14672	76956	24797	695	x	603	x	330	x	1927	1930	928	KP890739-45
T-010	22–02–1996	29–01–2011	39/F	<150	<150	<150	641	785	813	NA	525	x	880	1105	679	­
T-011	14–10–2004	29–01–2011	42/F	45583	7780	107965	823	559	489	533	575	x	1071	1191	1043	KP890746–61
T-012	20–09–2008	03–02–2011	30/F	<150	<150	x	1380	NA	1549	1964	x	x	1142	1071	x	­
T-013	20–04–2005	02–12–2011	25/F	438	3877	4528	856	580	816	718	502	724	1214	1424	1164	KT152635–37
T-014	28–10–2002	16–02–2011	35/F	2888	4578	x	553	484	376	436	425	x	1000	743	973	KT152638–42
T-015	11–05–2002	25–02–2011	36/F	542	3120	x	920	1000	820	880	830	x	1237	1097	1321	KT152643–47
T-016	22–10–2007	09–08–2011	38/F	1664	1401	x	730	x	653	x	x	x	1535	1366	x	KT152648–49
T-017	20–12–2006	27–09–2011	36/F	5598	x	x	504	332	325	x	x	x	2000	>2000	x	KT152650–52
T-018	16–03–2007	29–08–2011	34/M	6645	x	150244	519	563	483	452	446	x	1751	1732	1283	KT152653–56
T-019	31–05–2007	19–09–2011	39/M	63722	x	x	238	x	x	x	x	x	1379	x	x	KT152658
T-020	03–12–2008	11–12–2011	35/M	<150	489	x	459	x	x	x	x	x	>2000	x	x	KT152659
T-021	14–06–2008	14–10–2011	52/M	31410	x	x	676	651	641	x	x	x	884	861	x	­
T-022	20–07–2007	19–12–2011	38/M	6625	46553	x	624	522	558	488	x	x	>2000	>2000	x	KT152660–63
T-023	12–08–2004	23–02–2012	39/F	<150	x	x	987	x	x	x	x	x	1195	x	x	­
T-024	19–02–2007	28–02–2012	50/F	541	x	x	914	1179	1570	1574	x	x	1174	>2000	x	KT152664
T-025	11–06–2007	07–03–2012	38/F	1294	4369	x	789	950	693	x	x	x	1724	1968	x	KT152665
T-026	11–07–2007	15–03–2012	54/F	197	<150	974	907	x	1215	x	922	x	1345	1517	1748	­
T-027	29–04–2005	26–03–2012	36/F	<150	x	x	1195	630	x	x	x	x	767	x	x	­
T-028	25–05–2007	19–04–2012	28/F	162112	x	x	398	318	x	x	x	x	885	x	x	KT152666-68
T-029	31–12–2007	28–04–2012	30/F	1659	1504	x	618	694	599	545	x	x	1362	x	x	KT152669-70
T-030	22–12–2005	03–05–2012	42/F	284879	1E + 06	x	545	410	368	x	x	x	1352	x	x	KT152672-73
T-031	28–06–1999	09–06–2012	24/F	753	x	x	819	x	x	x	x	x	1091	x	x	­
(B) The Nellore cohort, *n* = 35																
Subject-ID	Enrolment date	Age/ Gender	Cell count		PVL											GenBank Accession No.
			CD4	CD8												Gag
INDO-SA-NLR2001	26–09–2011	23/F	608	1035	25149											­
INDO-SA-NLR2002	26–09–2011	32/F	589	927	26186											KT124420–21
INDO-SA-NLR2003	26–09–2011	69/M	1140	1192	548											KT124422
INDO-SA-NLR2004	26–09–2011	43/F	1012	1240	6070											KT124423–24
INDO-SA-NLR2005	26–09–2011	35/F	599	773	5713											KT124425–27
INDO-SA-NLR2006	26–09–2011	46/F	659	1185	662											KT124428
INDO-SA-NLR2007	26–09–2011	40/M	546	912	11986											KT124429–31
INDO-SA-NLR2008	26–09–2011	46/M	1289	1175	3687											KT124432–33
INDO-SA-NLR2010	26–09–2011	32/F	989	1497	251											KT124434–36
INDO-SA-NLR2011	26–09–2011	28/F	582	642	2704											KT124437
INDO-SA-NLR2012	28–09–2011	28/F	1087	1420	6070											KT124438–39
INDO-SA-NLR2014	28–09–2011	55/M	766	>2000	88645											KT124440–41
INDO-SA-NLR2015	28–09–2011	34/F	1019	>2000	21108											KT124442–43
INDO-SA-NLR2016	28–09–2011	29/F	631	1465	26363											KT124444–46
INDO-SA-NLR2017	28–09–2011	39/M	581	1050	8972											KT124447–49
INDO-SA-NLR2018	28–09–2011	35/F	825	1637	6493											KT124450–52
INDO-SA-NLR2019	28–09–2011	35/F	1085	1078	185											KT124453
INDO-SA-NLR2020	28–09–2011	38/M	570	1268	70025											KT124454–56
INDO-SA-NLR2022	28–09–2011	38/M	1489	1470	<150											KT124457
INDO-SA-NLR2023	28–09–2011	28/M	8.2	>2000	<150											­
INDO-SA-NLR2024	14–10–2011	30/F	984	1279	340											­
INDO-SA-NLR2025	14–10–2011	30/M	500	825	3423											KT124458–60
INDO-SA-NLR2026	14–10–2011	18/M	1015	1546	8733											KT124461–63
INDO-SA-NLR2028	14–10–2011	34/F	711	1409	24480											KT124464–65
INDO-SA-NLR2029	14–10–2011	35/F	1148	1290	<150											­
INDO-SA-NLR2030	14–10–2011	39/F	1185	1041	<150											­
INDO-SA-NLR2031	14–10–2011	50/F	1007	498	<150											­
INDO-SA-NLR2032	14–10–2011	45/M	671	821	9468											KT124466–68
INDO-SA-NLR2036	14–10–2011	27/M	860	1715	3332											KT124469–70
INDO-SA-NLR2037	14–10–2011	29/M	732	>2000	1505											KT124471–72
INDO-SA-NLR2038	31–10–2011	33/M	669	777	26542											KT124473
INDO-SA-NLR2039	31–10–2011	47/M	925	1111	4563295											KT124474–75
INDO-SA-NLR2040	31–10–2011	29/M	1181	978	<150											­
INDO-SA-NLR2041	31–10–2011	45/M	888	1237	56445											KT124476–77
INDO-SA-NLR2042	02–12–2011	26/F	1280	>2000	4797											KT124478

*Note*: Accession Numbers. KP890700-KP890762 and KT152677-686: Full-length *gag* sequences of six subjects (T002, T004, T006, T008, T009 and T011) at M0 time-point as determined using the plasmid clone sequencing strategy. The rest of the sequences were determined by direct sequencing of the PCR amplicons of the same subjects at the follow-up time-points

M: The month of sample collection at 6 month interval from baseline M0

X: Information not available

−: Failed PCR amplification

PVL: Plasma viral load (number of RNA copies/ml)

CD4 and CD8: cell count (cells/μl)

### The clinical procedures

A single vial of 20 ml of peripheral blood was collected from each participant at 6-month intervals during 2010–13. The blood samples were collected in EDTA vacutainers (Becton Dickinson, San Diego, USA) and were processed on the same day of collection. The PBMC and plasma samples were stored in 1 ml aliquots in a liquid nitrogen container and a deep freezer, respectively. Genomic DNA extracted from 200 μl of whole blood using a commercial DNA extraction kit (QIAmp Blood Mini Kit, Cat. No: 69504, Qiagen India, New Delhi, India). The CD4 T-cell count was determined using the BD FACSCount Reagent Kit (Cat. No: 340167, Becton Dickinson, San Jose, California, USA) and the BD FACSCount Control Kit (Cat. No: 340166) following the manufacturer’s instructions. The samples were analyzed on a BD FACS Calibur flow cytometer. The plasma viral RNA load was determined using the Abbott m2000rt Real-Time PCR machine (Abbott Molecular Inc. Des Plaines, IL, USA). All the methods were carried out in accordance with Institutional Ethics Committee for Human Research guidelines of the Indian Council for Medical Research (ICMR), New Delhi.

### RNA isolation and RT-PCR

RNA was extracted from 150 μl of plasma samples using a commercial Viral RNA isolation kit (NucleoSpin® RNA Virus, Ref. No. 740956.50, MACHEREY-NAGEL GmbH & Co. KG, Germany). In the case of the clinical samples that failed the PCR, an alternative kit was used to extract the viral RNA from 1 ml of plasma (the NucliSENS miniMAG nucleic acid extraction kit, Ref. No. 200293, BioMerieux, France). The complementary DNA (cDNA) was synthesized using random hexamers and a commercial kit (SuperScript® III Reverse Transcriptase, Cat. No: 18080–051, Invitrogen, Carlsbad, California, USA). The reaction vials were incubated at 25 °C for 10 min and 50 °C for 50 min. The reactions were terminated at 85 °C for 5 min followed by RNaseH treatment. The cDNA was used for the amplification of Gag and other viral gene segments.

### Amplification of Gag and the DNA sequencing

The full-length *gag* was amplified from the cDNA using a nested-PCR strategy and a commercial long-range PCR kit (XT-20 PCR system, Merck Genie, India) on the I-Cycler (Bio-Rad, California, USA). Furthermore, from a select subset of the participants, Gag p6, LTR, Protease, RT, RNaseH, Integrase and Env regions were also amplified. The details of the amplification and sequencing primers are summarized for *gag* in Table 2 and the rest of the gene segments in Table 3. The sequence of the V3-V5 envelope region (0.7 kb fragment, 7001–7667, HXB2 coordinates) of a few select subjects was determined using the HIV-1 Env Subtyping Kit (The NIH AIDS Research & Reference Reagent Program, Germantown, MD, USA).

**Table 2:** Primers for Gag amplification and sequencing

Amplification primers				
Primer Number	Description	Co-ordinates (HXB2) /Length	Sequence (5'–3')	Product length
Set I				
N420	EFP	683–709, 27	CTCTCGACGCAGGACTCGGCTTGCTGA	1712
N1476	ERP	2366–2395, 29	CTATCATTTTTGGTTTCCATYTTCCTGGC	
N1356	IFP	790–806, 27	AAGGAGACATATGGGTGCGAGAGCGTC	1516
N1435	IRP	2274–2306, 30	ATTTGGCCCCCTCGAGTTGAGACAAGRGGTCG	
Set II				
N420	EFP	683–709, 27	CTCTCGACGCAGGACTCGGCTTGCTGA	1832
N1634	ERP	2484–2515, 32	TTTCTTCCAATTATGTTGAYAGGTGTRGGTCC	
N1632	IFP	777–806, 30	TAGAAGGCTCGAGATGGGTGCGAGAGCGTC	1666
N1633	IRP	2413–2443, 32	ATRRGTATTTGATCATAYTGTCTTACTTTGAT	
Set III				
N1788	EFP	577–604, 29	GACTCTGGTAACTAGAGATCCCTCAGAC	2315
N1789	ERP	2862–2892, 35	ATGCATCA/CCCCACATCCAGTACTGTYACTG	
N1786	IFP	625–647, 28	ATCTCTAGCAGTGGCGCCCGAACAGGGAC	2211
N1787	IRP	2801–2836, 30	TGTGGAATTCCTAATTGRACYTCCCARAATCTG	
Set IV- p6 Gag				
N2301	EFP	1752–1778, 27	GTTGGTCCAAAATGCGAACCCAGATTG	1090
N2302	ERP	2813–2842, 29	GGGCCATCCATTCCTGGCTTTAATTTTAC	
N2304	IFP	1859–1885, 27	GCCACAAAGCAAGAGTGTTGGCTGAGG	549
N2305	IRP	2382–2408, 27	ACCTCCAATTCCTCCTATCATTTTTGG	
Sequencing primers				
N1619	Forward Primers	1040–1056, 17	GGCCATTGACAGAAGA	
N1620		1609–1626, 18	TTGTATGTAGGATCTGA	
N1621		2092–2107, 16	TGCCCACACTAATGATG	
N1812		2035–2058, 25	TGTGGAAAGGAAGGACACCAAATG	
N1489		1302–1326, 25	TGTTTACAGCATTATCAGAAGGAGC	
N1622	Reverse primers	1835–1818, 18	GCTGTCATCATYTCTTCT	
N1623		1329–1313, 17	GGTGGCTCCTTCTGATA	
N1624		910–895, 16	GCTCCCTGCTTGCCCA	
N1490		1826–1849, 24	CACTCCCTGACATGCTGTCATCAT	

EFP- External forward primer, ERP-External reverse primer, IRP- Internal reverse primer, IFP-Internal forward primer, Reverse primers have been presented as reverse complement

**Table 3:** Primers for Pol and LTR amplification and sequencing

Amplification primers				
Primer Number	Description	Co-ordinates (HXB2) /Length	Sequence (5'–3')	Product length
Protease				
N1636	EFP	1978–2006, 29	AAGGAAGGGCACCCAGCCAGAAATTGCAG	864
N1637	ERP	2813–2842, 30	GCGGGATGTGGTATCCCTAATTGAACTTCC	
N1638	IFP	2075–2103, 29	GACAGGCTAATTTTTTAGGGAARATTTGG	530
N1639	IRP	2577–2605, 29	GGGCCATCCATTCCTGGCTTTAATTTTAC	
RT				
N1641	EFP	2368–2395, 28	CAGGAAGATGGAAACCAAAAATGATAGG	2114
N1642	ERP	4455–4482, 28	CTATATATCCACTGGCTACATGAACTGC	
N1643	IFP	2489–2509, 21	TACACCTGTCAACATAATTGG	1816
N1644	IRP	4285–4305, 21	AATCACTAGCCATTGCTCTCC	
RNaseH/Integrase				
N2244	EFP	3628–3660, 33	CCCACACTAATGATGTAAAACAGTTAACAGAGG	1659
N2245	ERP	5257–5287, 31	CCATGACCCAAATGCCAATCTCTTTCTCCTG	
N2246	IFP	3738–3765, 28	ATGGGAAACATGGTGGACAGACTATTGG	1476
N2247	IRP	5185–5214, 30	TGGGATGTGTACTTCTGAACTTAYTTTTGG	
LTR				
N698	EFP	1–34, 34	TGGAAGGGTTAATTTACTCYMAGAAAAGRCAAGA	555
N1032	ERP	538–556, 19	TAGAGCACGCAAGGCAAGC	
N558	IFP	1–43, 43	TGGAAGGGTTAATTTACTCTAAGGAAAGGCAAGAGATCCTTG	431
N1204	IRP	411–432, 21	CTTATATGCAGGATCTGAGGG	
Sequencing primers				gene
N1645	Forward Primers	2620–2635, 16	GGCCATTGACAGAAGA	RT
N1646		3108–3124, 17	TTGTATGTAGGATCTGA	
N1647		3626–3642, 17	TGCCCACACTAATGATG	
N1648	Reverse primers	3870–3886, 17	GCTGCCCCATCTACATA	
N1649		3345–3362, 18	GTAAATCTGACTTGCCCA	
N1650		2889–2906, 18	GGGAACTGAAAAATATGC	
9 M	Forward Primers	4145–4167, 21	CCTGTCATGGGTACCAGCACA	RNaseH/Integrase
10 M		4664–4687, 24	CCAAAGTCAGGGAGTAGTAGAATC	
28 M	Reverse primers	4957–4978, 22	ACTACTGCCCCTTCACCTTTCC	
29 M		4380–4401, 22	GACTGCAGTCTACTTGTCCATG	

Protease and LTR were sequenced using amplification primers

The reaction conditions for the Gag amplification in both the rounds of the nested PCR were 94 °C for 2 min for one cycle and then 35 cycles, each cycle consisting of melting at 94 °C for 30 s, annealing at 50 °C for 50 s, and extension at 72 °C for 2 min, followed by final extension at 72 °C for 5 min. Two μl of the first-round PCR products were transferred to the second-round PCR to amplify the complete *gag* sequence. The details of the primers and the amplified products are summarized in Table 2. Carryover contamination was prevented by adherence to strict procedural and physical safeguards that included reagent preparation and PCR setup, amplification, and post-PCR processing of samples in separate rooms. The PCR products were purified using a commercial DNA purification kit (Cat. No. YDF100, Real Biotech Corporation, Taiwan) and subjected to sequencing. The sequencing was performed on ABI PRISM® 3130xl Genetic Analyser (Applied Biosystems, Illinois, USA) using multiple internal sequencing primers. The sequences are available from GenBank under the accession numbers KF578465-KF578467, KT124420-KT124478, KT152633-KT152672, and KP890700-KP890762.

### Phylogenetic and sequence analyses

Phylogenetic analysis was performed using the reference sequences of HIV-1 group M available from the Los Alamos database (http://www.hiv.lanl.gov/). Sequences were manually edited using Bio-Edit software version 7.0.5.3. Multiple sequence alignments were performed using ClustalW in the BioEdit software (version 7.0.5.3). The phylogenetic tree was constructed using the neighbor-joining method (Kimura two-parameter model) in 1,000 bootstrapped data sets, using the Molecular Evolutionary Genetics Analysis (MEGA) software version 5 [30]. The evolutionary distances were computed using the Maximum Composite Likelihood method and are in the units of the number of base substitutions per site. All the positions containing gaps and missing data were eliminated. The final dataset contained a total of 1,632 nucleic acid positions. Additionally, genetic subtype characterization of the *gag* sequences was performed using the REGA HIV-1 Subtyping Tool version 2.0, available at the BioAfrica site (http://www.bioafrica.net/rega-genotype/html/) and the recombinant identification tool available at the Los Alamos HIV Sequence Database (http://hiv.lanl.gov/content/index). Every individual gene sequence was subjected to a BLAST analysis against the laboratory sequence database to confirm authenticity. All the other HIV-1 *gag* sequences required for the analysis were retrieved from the Los Alamos HIV Sequence Database (http://hiv.lanl.gov/content/index).

To evaluate the global prevalence of the PTAP duplication, the Los Alamos National Laboratory (LANL) HIV sequence database was searched for global HIV-1 *gag* sequences for subtype A, B, C, D, F, G, CRF01_AE, CRF02_AG and CRF07/08_BC as of October 2015. Of note, the partial sequence insertions not duplicating the core PTAP motif or amino acid insertions not related to PTAP were not included in the analysis. *In silico* analysis was performed to understand the prevalence of PTAP duplication. Further, to evaluate the profile of PTAP duplication over the past 30 years, subtype B and C sequences were downloaded from the LANL sequence database excluding the problematic sequences and acquiring one sequence per patient. Of note, for many sequences, the information regarding the date of isolation of the clinical sample was not available from the database. We, therefore, considered the date of sequence submission instead for the analysis. It should be noted that there could be a significant delay between the actual date of sample collection and sequence submission. Amino acid sequences downloaded in the FASTA format were categorized into five groups representing five time periods, beginning with the year of the first sequence deposited till 2015. The regression analysis was performed using GraphPad Prism version 5.02 to examine the trend of PTAP motif duplication over the period of past 30 years.

## Results

### PTAP duplications of longer sequence length are inserted at a higher frequency in subtype C

**Fig. 2:**
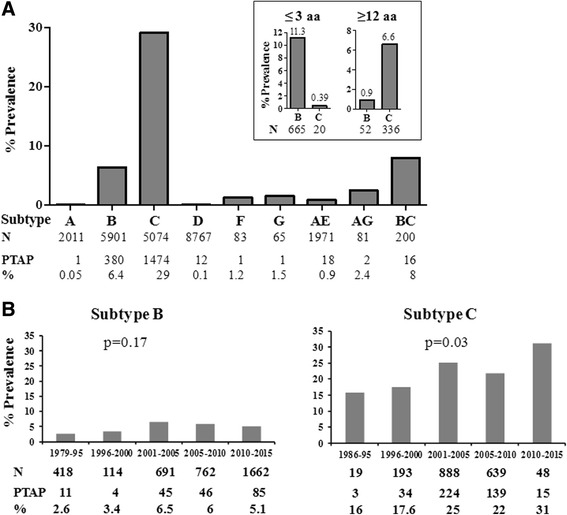
**a** The relative prevalence of PTAP insertion in HIV-1 subtypes. Full-length *gag* sequences belonging to the various genetic subtypes of HIV-1 were downloaded from the LANL HIV sequence database. The subtype identity, total number of *gag* sequences used in the present analysis and the number of viral isolates containing the PTAP motif duplication are shown. The percent prevalence of the PTAP motif duplication under each genetic subtype is depicted. Subtype A represents subtypes A, A1, and A2 and subtype F represents subtypes F, F1, and F2. BC includes 7 different BC recombinants (CRF_07: 8/98, CRF_08: 7/83, CRF_31: 1/3, CRF_60: 0/3, CRF_61: 0/4, CRF_62: 0/3 and CRF_64: 0/6) with PTAP duplication. Inset: The number and percentage of viral sequences containing insertions of 3 aa or shorter and 12 amino acids or longer are depicted for subtypes B and C. **b** The relative prevalence of double-PTAP strains in HIV-1 subtype B and C over the past 30 years. Only those sequences that contained the core PTAP motif (four amino acids or more) were included in the analysis. Subsets of the full-length *gag* sequences belonging to HIV-1 subtype B (3,647 sequences) and C (1,787 sequences) were used in the analysis. Only one sequence per subject was included in the analysis. Sequences were categorized into 5-year periods starting from 1979 (subtype B) or 1986 (subtype C) to 2015. The subtype identity, total number of *gag* sequences used in the analysis and the number of viral isolates containing the PTAP motif duplication are shown. The percent prevalence of the PTAP motif duplication under each genetic subtype is depicted. The increasing trend of PTAP duplication in subtype C was found to be statistically significant (*p* < 0.05) using the regression analysis

In the present manuscript, we refer to all the viral strains that contain a complete PTAP motif duplication in Gag p6 as the double-PTAP strains and those that lack such duplication as the single-PTAP strains. The double-PTAP strains must contain a second and intact PTAP motif comprising of the four core amino acids with or without the additional flanking amino acids. To examine the nature of the PTAP sequence insertion in HIV-1 Gag p6 in different HIV-1 genetic subtypes, we analyzed a total of 17,769 full-length HIV-1 Gag sequences available from the Database. Eleven percent of the sequences (1,997) contained insertions that constituted the duplication of the PTAP motif. The analysis identified two important characteristics of subtype C. The frequency of the sequence duplication and the length of sequence duplication, both was found significantly higher in subtype C as compared to other subtypes (Fig. 2). Twenty-nine percent of the sequences (1,474 of 5,074) of subtype C contained the complete duplication of the PTAP motif (Fig. 2a). A considerably smaller proportion (380 of 5,901; 6.4%) of subtype B strains also contained a complete PTAP duplication. In contrast, only a low frequency of subtype D sequences (12 of 8,767; 0.1%) contained the PTAP duplication. All the other HIV subtypes collectively contained less than 5% of sequences characterized by the PTAP duplication. The BC recombinant HIV-1 strains represented 8% of sequences containing the PTAP duplication. The HIV-1 groups N, O, and P contained few sequences to permit a meaningful sequence analysis.

Furthermore, the length of the PTAP motif duplication was quite heterogeneous with some of the insertions being as long as 16 amino acid residues. To understand if the length of the sequence insertion has any association with the nature of viral subtype, the sequences of subtypes B and C were compared, as only these two subtypes contained sufficient numbers. In subtype C, while 7% (357/5,074) of Gag p6 sequences contained PTAP insertions 12–16 amino acids or longer, only 0.39% (20/5074) of the sequences contained sequence duplication of three amino acids or less (Fig. 2a). In contrast, in subtype B, only 1% of the sequences (52/5,901) contained PTAP insertions of 12–16 amino acids and as many sequences as 11.2% (665/5901) contained insertions of three amino acid or less. Collectively, the above analysis is indicative that subtype C has the propensity to introduce PTAP sequence duplications at a higher frequency, and the duplication length is typically longer.

Additionally, to understand if the prevalence of the PTAP duplication has been undergoing a variation over the years, we categorized the full-length *gag* sequences of subtypes B and C into 5-year phases starting from 1979 (subtype B) or 1986 (subtype C) up to 2015 (Fig. 2b). In subtype B, the percent prevalence of the PTAP duplication strains increased between phases 1996–2000 to 2001–2005 from 3.4 to 6.5% and remained stable after that. A comparable trend was seen in the context of subtype C but at a higher prevalence. Between phases 1996–2000 to 2001–2005, the percent prevalence of the PTAP duplication strains of subtype C increased from 17.6 to 25% and increased further to 31% prevalence in the final phase of 2010–2015 although the sample size for the last phase is indeed small. Using the regression analysis to understand the trend, we observed that the increasing trend of PTAP duplication in subtype C is statistically significant (*p* = 0.03) unlike in subtype B (*p* = 0.17) where no such trend was seen. Thus, unlike in subtype B where the prevalence of the PTAP duplication appears to have been stabilized at the global level, the prevalence of PTAP duplication appears to be increasing further in subtype C, which must be confirmed in future studies.

### The length of the PTAP motif duplication varies between the primary viral isolates but remains constant through the chronic phase

The sequence analysis performed above confirmed the global predominance of the PTAP motif duplication in subtype C Gag sequences. However, the disease status of the subjects, and their anti-retroviral therapy status and its influence on the PTAP motif duplication are not known. Additionally, the profile of the PTAP sequence duplication and its evolutionary stability has not been examined in a longitudinal study especially in the context of subtype C infection. To fill this gap, we monitored two different southern Indian clinical cohorts, consisting of 65 drug-naïve subjects; in a 2 to 3 years follow-up spanning 2010–2013 with a repeat blood sampling every 6 months. All the subjects belonged to the chronic stage of the viral infection and were infected with subtype C reportedly by heterosexual transmission. The clinical profile of all the 65 study subjects has been documented (Table 1).

**Fig. 3:**
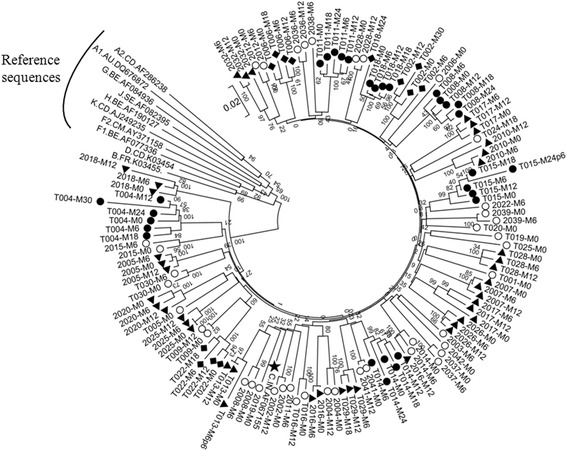
The phylogenetic analysis of the *gag* sequences using the neighbor-joining method. Full-length *gag* sequences (*n* = 126) derived from 50 subjects were used in the analysis. For two samples (T013-M6p6 and T015-M24p6), only *gag p6* could be amplified. Filled circles, filled diamonds and filled triangles represent the subjects from whom sequences were available at ≥ five (*n* = 6), four (*n* = 3) and three (*n* = 13) different time points, respectively. Open circles represent all the other subjects (*n* = 28), from whom sequences at less than three time-points were available. The reference sequences of *gag* from various genetic subtypes - A, B, C, D, E, F, G, H, J, and K were downloaded from the Los Alamos HIV sequence database. The star represents subtype C reference sequence. After the gap stripping, there were 1,632 nucleic acid positions in the final dataset. The percentage of replicate trees in which the associated taxa clustered together in the bootstrap test (1,000 replicates) are shown next to the branches. The tree is drawn to scale, with branch lengths in the same units as those of the evolutionary distances used to infer the phylogenetic tree. The evolutionary distances were computed using the Maximum Composite Likelihood method and were in the units of the number of base substitutions per site

**Fig. 4:**
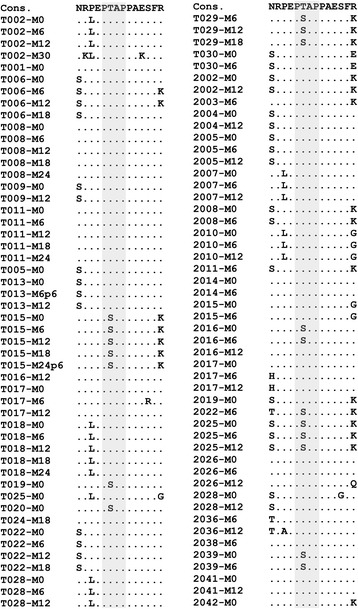
Multiple sequence alignment of the PTAP region. One hundred sequences derived from 42 subjects were aligned with the subtype C consensus (Cons.) amino acid sequence. In the alignment, the dots represent sequence identity. The PTAP core motifs are highlighted using the gray shade

**Fig. 5:**
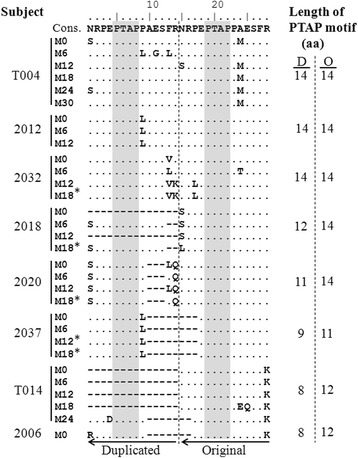
Multiple sequence alignment of the PTAP sequences of eight primary viral isolates containing PTAP duplication. The sequences were derived from the plasma viral RNA collected at multiple longitudinal time-points and by sequencing the PCR fragments. In three subjects (T004, 2012, 2032), the PTAP motif duplication involved the duplication of 14 amino acids. In the other five subjects, the duplicated sequence length was shorter containing 12 (2018), 11(2020), 9 (2037) or 8 (T014, and 2006) residues. Of note, in all the sequences, the ‘PTAP’ core motif was intact in the original and the duplicated sequences. Note that in two subjects (2014 and 2018) a mixed infection of single- and double-PTAP viral strains could be seen by conventional sequencing. In subject T014, PTAP duplication was observed only at a single time-point M24. For subject 2006, the plasma sample was available only at the baseline. The vertical dashed line demarcates the original and the duplicated PTAP motifs. The arrows indicate the direction of the RT polymerization. Cons: The consensus subtype C *gag* sequence of 14 amino acids. The same 14 amino acid consensus sequence was used twice in the analysis for convenience. *For these five samples, only *gag p6*, not the full-length *gag*, was amplified. These sequences have not been deposited in the Genbank and are not part of the phylogenetic tree presented in Fig. 3. D: the duplicated PTAP motif, O, the original PTAP motif

Using a nested PCR, we could successfully determine the full-length *gag* sequence from 50 of the 65 subjects either from the plasma viral RNA or the genomic DNA extracted from whole blood. In a phylogenetic analysis, all the primary *gag* sequences clustered together and with the reference subtype C viral sequences confirming their phylogenetic identity (Fig. 3). Of the total 126 *gag* sequences generated from the 50 subjects, 100 sequences derived from 42 subjects lacked a sequence duplication of any kind, thus, representing the wild type single-PTAP *gag* profile (Fig. 4). The PTAP elements consisting approximately of 14 amino acid residues of the 100 Gag sequences were aligned (Fig. 4). Several of the sequences contained nucleotide insertions or deletions of variable length including single amino acid variations in different regions of Gag. In two subjects (T019 and 2010), sequence insertions were found at the N-terminal of Gag p6, but these duplications were not related to the PTAP motif. The PTAP motif duplication was found in the remaining eight of the 50 subjects (T004, T014, 2006, 2012, 2018, 2020, 2032, and 2037). Thirty-one sequences from the eight subjects were aligned in the PTAP region consisting of a 14 amino acid window (Fig. 5). A sequence of 14 amino acids (NRPEPTAPPAESFR, the four core residues underlined) representing subtype C consensus PTAP motif consisting of the four core residues and the ten flanking residues was used as references in the multi-sequence alignment. Several important observations could be made from the sequence analysis regarding PTAP duplication in the eight subjects. First, the length of sequence duplication was variable in the primary viral isolates. The length of the original PTAP motif (right side of the dashed line, Fig. 5) and that of the duplicated PTAP motif (left side of the dashed line, Fig. 5) both vary in the length of the sequences. For a simple representation of these differences in the PTAP motifs, we use a formula consisting of two figures. to represent each viral isolate. The right and left-hand numbers in the formula represent the number of amino acid residues in the original and duplicated PTAP motifs in Gag p6 of the viral isolate, respectively. Using this formula, we found that three of the eight subjects (T004, 2012, and 2032) contained an original PTAP motif of 14 residues and a duplication of 14 amino acids thus representing a configuration of 14 + 14. In contrast, subjects 2018 contained a PTAP configuration of 12 + 14 representing an original PTAP motif of complete 14 amino acid length but a duplicated PTAP motif shorter by two amino acids and 2020 contained a PTAP configuration of 11 + 14 representing a duplicated PTAP motif shorter by three amino acids. Subject 2037 contained a 9 + 11 configuration while the other two subjects T014 and 2006 an 8 + 12 configuration of PTAP duplication. Second, regardless of the differences constituting the original or duplicated PTAP motifs, in all the subjects at all the time points, the ‘core PTAP motif’ is invariably conserved. Additionally, the aspartic amino acid residue (E) located immediately upstream of the core motif is also invariably conserved in the original as well as the duplicated sequences alluding to the functional importance of these five (EPTAP) residues. Additionally, in an analysis of sequences downloaded from the extant database also, we observed that the upstream aspartic amino acid residue is highly conserved. Only 34 of 1,804 (1.8%) subtype C sequences demonstrated variation at this residue (single sequence per subject was included in the analysis, data not shown). The variations observed between the original, and the duplicated sequences were exclusively mapped to the other nine flanking residues of the 14 amino acid motif. The highly preserved ‘EPTAP’ motif is also seen in the viral strains containing a PTAP motif duplication of shorter length. Third, in all the viral strains, the duplicated PTAP motif typically demonstrates variation at least in one residue as compared to the original PTAP motif. Thus, a sequence variation between the original and duplicated PTAP motifs appears more common and necessary. Between the original and the duplicated PTAP motifs, the former is usually genetically more homologous to subtype C consensus sequence. Thus, the duplicated PTAP motif demonstrates a sequence variation of a higher order. Fourth and importantly, the length of the duplicated PTAP sequence remained constant at the follow-up time points in each subject suggesting the stability of the sequence length once the motif is duplicated. In subject T004, for instance, the sequence of the duplicated motifs remained constant consisting of 14 residues at all the six different time points spaced 6-month apart (M0, M6, M12, M18, M24, and M30). Additionally, the duplicated sequence may undergo further variation in the amino acid sequence (compare time points M0, M6 and M12 of subject T004. Likewise, different time points of subjects 2032 and 2020), but not in the length of the motif. This observation suggested that in the chronic phase of the viral infection, once the founder viral strains were established possibly in the acute phase, the length of the duplications remain stable although the sequence diversity within and between the duplicated and the original 14 amino acids is highly variable. Lastly, at least in two subjects (2018 and 2014), the presence of the single-PTAP as wells as double-PTAP viral strains was detected suggesting a possible mixed infection. Of note, the conventional PCR amplicon sequencing strategy employed here is limited by the inability to detect minority viral strains if they are not represented at least at a prevalence of 20% [31]. Thus, it remains a possibility that other subjects also might contain the single-PTAP viral strains, but these minority species were not visible in the conventional sequencing that we employed here. In summary, the PCR fragment sequence strategy identified that the original PTAP sequence was relatively conserved among the subjects, but when any part of the sequence comprising of the PTAP core motif is duplicated, the amino acid residues flanking the PTAP core demonstrated a higher magnitude of genetic variation.

### In the six study subjects, PTAP duplication was not associated with the drug-resistance mutations

To find a possible association between PTAP duplication and drug resistance, primary or transmitted, we determined the sequences of the *pol* sequence (Protease, RT, RNase H, and Integrase; coordinates 2,253 to 5,096 as in HXB2) of the viral strains from all the six subjects of our cohort containing PTAP duplications. We could successfully amplify the protease from all the six subjects (Accession numbers: KT124484-KT124490). The RT (Accession numbers: KT124479-KT124483) and Integrase (Accession numbers: KT428977-KT428981) regions could be amplified from only five subjects. The sequences were subjected to drug resistance analysis using the HIV drug resistance database at the Stanford University (http://hivdb.stanford.edu/). This analysis failed to find any mutations associated with drug resistance in any of the viral enzymes in these six subjects. Thus, this analysis, on the one hand, confirmed the drug-naïve status of the six subjects and on the other hand, ruled out ART as a cause for the appearance of PTAP duplication in these subjects. The immune pressure in these subjects could be a selection pressure that may have mediated the PTAP sequence duplication. To understand if PTAP duplication is associated with the sequence insertions in other regions of the viral strains, we determined the sequences of the *LTR* and *env* from the six subjects containing the PTAP duplication. *Env* and *LTR* could be amplified from four (ascension no: KP683336-KP683342) and five (ascension no: KP683343- KP683348) of the six subjects, respectively. We did not find any sequence insertions or other modifications in the V3 loop of the envelope or the viral promoter (data not shown).

### The PTAP sequence duplication in a South-African clinical cohort

**Fig. 6:**
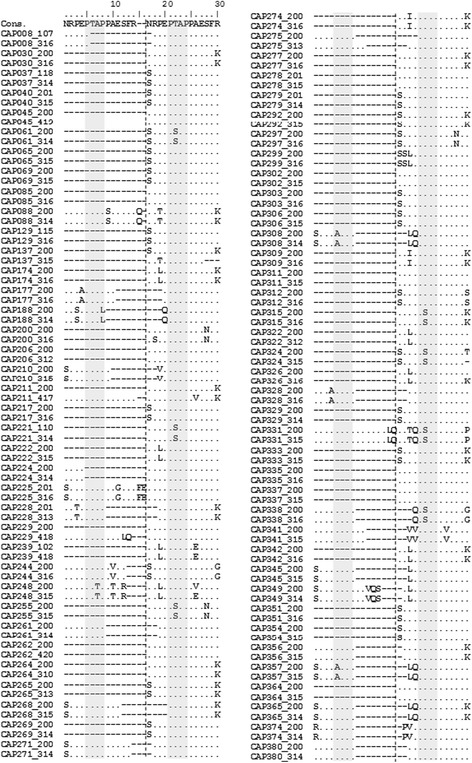
Multiple sequence alignment of the PTAP region amino acid residues of the CAPRISA Cohort. One hundred and fifty sequences derived from 75 subjects were aligned with the subtype C consensus (Cons.) sequence. In the alignment, the dots represent sequence identity and the dashes sequence deletion. The PTAP core motifs are highlighted in the gray shade. The vertical dashed line demarcates the original (right side) and the duplicated (left side) PTAP motifs. The consensus subtype C *gag* sequence of 14 amino acids was used twice in the analysis for convenience

**Fig. 7:**
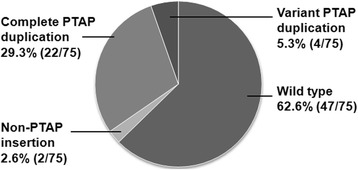
The pie chart depicts the profile of the sequence insertion in Gag p6 of the CAPRISA cohort. The 150 *gag* sequences derived from 75 subjects may be classified into four categories based on the nature of PTAP duplication; the sequences not containing insertions of any nature (wild type, 96/150, 64%), variant PTAP duplication (8/150, 5.3%), non-PTAP insertions 4/150, 2.6%), and complete PTAP duplication 42/150, 28%). The slices in the pie chart, however, represent the subjects, not sequences, to avoid confusion regarding the proportion of complete PTAP duplication between subjects versus sequences. The proportion of complete PTAP duplication will be 29.3 or 28% depending on whether the subjects (22 of 75) or sequences (42 of 150), respectively, are considered

As in India, the viral epidemic in South Africa is dominated by HIV-1 subtype C. We had access to the full-length *gag* sequences of the CAPRISA clinical cohort in the city of Durban and rural region of Vulindlela (the Acute Infection Study Team, CAPRISA, Durban), South Africa. The clinical profile and the study objectives of the cohort have been reported previously [32, 33]. The *gag* sequences of subtype C origin were derived from 75 subjects, constituting a subset of the CAPRISA 002 cohort. All the 75 subjects were drug-naïve, in the chronic phase of the infection, and recruited during 2004 to 2010. Access to the CAPRISA clinical cohort offered an opportunity to examine the prevalence and evolution of PTAP duplication in a different clinical cohort dominated by subtype C. A total of 150 subtype C p6 *gag* sequences, two from each subject, were available for the analysis. The first sequence from each subject was drawn from the acute phase of the infection at the time of screening or enrollment while the second sequence at a later time point eight to 25 months post-recruitment, representing the chronic phase of the infection. All the 150 p6 *gag* sequences were evaluated for sequence insertion of any type in the PTAP motif region of p6 and aligned with a subtype C consensus sequence generated using the sequences derived from the Los Alamos HIV Sequence Database, 2004 (Fig. 6). The analysis identified sequences of four different kinds in the 75 subjects depending on the nature of PTAP duplication. Of the 150 sequences, 64% (96/150) did not contain a sequence insertion of any kind in the PTAP motif region thus, representing the wild type profile. Of the rest of the 54 sequences, four sequences derived from two subjects (CAP008 and CAP224) lacked the duplication of the PTAP motif but contained an insertion of a sequence string located immediately upstream of the PTAP motif. Of the remainder of the 50 sequences, 42 sequences (28%, 42/150) contained a complete duplication of the PTAP core motif (Fig. 7). In the remaining eight sequences derived from four subjects (CAP188, CAP248, CAP308, and CAP357), the duplicated PTAP motif was mutated to PTAL, ATAP or PTTP motifs as also observed in the LANL database. Each of the mutated motifs differed from the PTAP motif at a single amino acid residue. It is not known if such variations are biologically functional. We did not find such variations in the duplicated PTAP motif in the Indian clinical cohort of the present study probably given the small sample size, only eight viral strains. The insertion length in the 42 sequences containing the complete PTAP duplication ranged from 4 to 16 amino-acids in the South African cohort. Importantly, of the 42 *gag* p6 sequences derived from 22 subjects that contained a complete PTAP duplication, in 20 subjects, the duplication was observed at both the time points of sample collection, at baseline and the follow-up time point. In two subjects (CAP211 and CAP229), the duplication was found only at the follow-up time point but not at baseline. Since a conventional Sanger sequencing strategy was used to examine the viral species in all these samples, it is not known if the non-detection of the PTAP variant viral strains at earlier time points from CAP211 and CAP229 could be ascribed to the limitations in sensitivity. Collectively, the data demonstrated a prevalence of 29.3% (22 of 75 subjects) PTAP sequence duplication in the drug-naïve population of the South African subtype C viral strains, thus, independently confirming our observations. The present analysis combined with that of the published reports of subtype C collectively ascertains that (1) there is significantly higher representation of the PTAP motif duplication in subtype C as compared to the other subtypes, (2) the mean length of the PTAP sequence duplication in subtype C is significantly larger.

## Discussion

Our analysis found that PTAP duplication was significantly higher in subtype C compared to other subtypes. We identified that only a small proportion of global subtype B sequences (52/5901, 0.9%) contains a sequence insertion of 12 amino acids or more leading to the complete PTAP motif duplication whereas in subtypes A and D the complete PTAP sequence duplications is extremely rare. In stark contrast, 29% of the global subtype C sequences contained insertions leading to complete PTAP motif sequence duplication (1,474/5,074) (Fig. 2a). Thus, in subtype C, sequence insertions in PTAP domain occur at a higher frequency, most likely even in the absence of the ART administration which has been reported to commonly augment the frequency of the complete duplication of the PTAP motif [22, 28, 34]. The high prevalence of complete duplication of the PTAP motif in subtype C, especially in drug-naïve subjects, suggests the biological significance of this phenomenon for subtype C. In this backdrop, through the present study, we attempted to investigate the frequency of PTAP duplication in subtype C viruses derived from primary clinical isolates of drug-naïve subjects of India.

The double-PTAP viral strains represented 16% (8/50) of prevalence in our clinical cohorts. The prevalence of the PTAP variant strains in three other cohorts of subtype C is significantly higher than that of our cohort. In the well-characterized CAPRISA clinical cohort of the acute phase infection of subtype C, the PTAP variant viral strains represent 29.3% (22/75) of the primary infections (Fig. 7) and 30% (212/706) in a South African clinical cohort [35]. Further, in a cross-sectional analysis of a Brazilian clinical cohort, the double-PTAP strains represent 23% (52/228) of the infections in the drug-naïve subjects and 54% (33/61) in drug-exposed subjects [28]. The epidemic of India is believed to be younger by at least a decade as compared to that of the Africa partly explaining the differences in the frequency of PTAP duplication between the two clinical contexts.

Our study also demonstrates that subtype C is endowed with a superior ability to duplicate longer stretches of amino acid residues permitting the complete duplication of the PTAP motif. The length of complete PTAP duplication in our cohort ranged from 8 to 14 amino acids. Our analysis for the first time showed that the length of PTAP duplication was highly stable over a period of 2–3 years of the chronic phase. Thus, it appears that once a PTAP duplication of a defined length is acquired, possibly in the early phases of the viral infection, a variation in the sequence, but not the length, of the duplicated sequence is permitted at the subsequent time points. The inferences drawn from the South African and Indian sequences regarding the length variation of the PTAP duplication are consistent with each other.

The data presented here are limited by the sample size and our inability to draw a correlation between the presence of double-PTAP viral strains and disease progression given the lack of information on the plasma viral load of the subjects and the date of infection. Further, we also could not perform stringent statistical evaluations on the confounding variables such as the HLA profile, CD4 counts, and viral load, etc. Nevertheless, all subjects recruited into the study were drug-naive, believed to have acquired the virus through heterosexual transmission, and were free from opportunistic infections and AIDS-related symptoms. Given these limitations, our results can be considered as inferential evidence suggesting a positive evolutionary selection of the variant viral strains in drug-naive subjects. Nonetheless, the prevalence of double-PTAPviruses (8/50, 16%) in drug-naive subjects from two clinical cohorts of India and in a cohort from South Africa (22/75 subjects, 29.3%, the Acute Infection Study Team, CAPRISA, Durban) in a longitudinal study, suggests the progressive expansion of variant viral strains in subtype C. The high prevalence of the Double-PTAP viral strains in subtype C has been corroborated by the recent studies from Brazil [28] and South Africa [35].

Subtype C appears to exploit the phenomenon of sequence insertion as a powerful strategy to duplicate sequence motifs of biological significance to gain replication advantage. We previously demonstrated that in the viral promoter, only subtype C viral strains demonstrate a potential to duplicate the NF-κB binding site while all the HIV-1 subtypes are capable of duplicating the upstream RBEIII binding site including subtype C [4]. The duplication of the 21 base pairs that constitute the additional NF-κB motif in the viral LTR is highly faithful without a sequence variation in the duplicated sequence. The PTAP motif duplication differs from that of NF-κB motif duplication in being highly variable especially in the flanking nine residues. Unlike the DNA sequences in the viral promoter, the amino acid sequences in a viral protein are prone to immune detection and hence must undergo rapid variation to evade the immune response. The selection pressure to avoid immune response perhaps could explain why the PTAP motifs, the original as well as the duplicated sequences, are highly variable when the duplicated NF-κB motif is highly conserved in subtype C. The NF-κB duplication, and PTAP duplication in subtype C also differ from each other in another property. The ability to duplicate the NF-κB motif is an exclusive property of subtype C, not manifested by other HIV-1 subtypes [5]. In contrast, both subtypes B and C can duplicate sequences in the PTAP domain. Thus, PTAP duplication is not an exclusive property of subtype C although subtype C appears to be significantly superior in the ability to duplicate PTAP. Only 0.9% of subtype B sequences deposited in the databases contain PTAP duplication of 12 amino acids, or more whereas 6.6% of subtype C strains contain such large size duplications (Fig. 2a, inset). The near absence of the complete PTAP motif duplication in other subtypes, primarily in A and D for which a large number of sequences are available in the databases, is quite intriguing. Lastly, the length of the sequence duplication to constitute the additional NF-κB motif appears to be fixed, 21 bases, and invariable in subtype C [5]. In contrast, the sequence length of PTAP duplication is highly variable in both subtypes B and C ranging from 4 to 16 amino acids [28, 35] (Fig. 5). Because of the complexity of viral quasispecies, we sequenced *gag* from the genomic DNA (data not shown) of the selected subjects and observed the PTAP duplication in the proviral compartment also. Analysis of sequences from the proviral compartment will not be the representative of the quasispecies circulating in the plasma, hence it was important to capture the presence of circulating virions using plasma viral RNA.

It would be critical to know if the PTAP variant viral strains are likely to expand in the population in the coming years like the NF-κB variant viral strains have been doing currently. At the population level, the relative success of an emerging variant viral strain primarily depends on the difference in the plasma viral loads of the two divergent variant strains, especially in a mixed infection where the single-PTAP and double-PTAP viral strains coexist in the same host. It is not known if PTAP duplication in Gag p6 modulates the infectivity properties of the envelope in any manner. Especially, in the context of a mixed infection, the PTAP divergent viral strains are not likely to differ from each other with respect to the envelope considering the enormous magnitude of viral recombination in vivo. The PTAP divergent viral strains sharing the same envelope, therefore, are expected to maintain identical biological properties, including cell tropism, the preferred route of transmission, and target cell populations. In a mixed infection, a viral variant such as the strain containing a double-PTAP motif represented by a profoundly higher magnitude of plasma viral load (unpublished data) is likely to be transmitted at a significantly higher rate to the new host. If this prediction holds true, the PTAP variant viral strains are expected to expand rapidly replacing the single-PTAP viral strains in the coming years.

Furthermore, to address the question whether the PTAP motif duplication is associated with the expansion of the variant viral strains, we downloaded the available full-length Gag sequences of subtypes B and C from the LANL HIV sequence database. Using these sequences (3,647 and 1,787 representing subtypes B and C, respectively), we asked if the percentage prevalence of the viral strains containing the duplication increased over the past 30 years when stratified into 5-year phases starting from 1979 (Fig. 2b). Given the large sample size, the sequences are expected to be representative of the viral prevalence in the natural infection. In subtype B, the percent prevalence of the double-PTAP strains doubled between phases 1996–2000 to 2001–2005 from 3.4 to 6.5% and remained stable after that. A comparable trend was seen in the context of subtype C but at a higher prevalence. Between phases 1996–2000 to 2001–2005, the percent prevalence of the double-PTAP strains of subtype C increased from 17.6 to 25% and increased further to 31% prevalence in the final phase of 2010–2015. Importantly, a progressively increasing prevalence of the double-PTAP viral strains was evident in subtype C although the sample size available at the latest phase 2010–2015 is limited (Fig. 2b). Thus, it appears that unlike in other HIV-1 genetic families, in subtype C alone, the PTAP duplication appears to be associated with the global expansion of this HIV-1 subtype. It is necessary to monitor the expansion rates of the diverse PTAP and 4-κB viral strains in the coming years.

## Conclusions

The most significant finding of the present analysis is that we demonstrated for the first time that the length of PTAP duplication, but not the sequence within the duplicated motif, is stable in the chronic phase of HIV-1 infection. We also showed that the frequency of PTAP duplication is considerably higher in subtype C as compared to other viral subtypes. The subtype C epidemic in India is believed to be younger by at least a decade as compared to that of Africa. This difference might partly explain why the frequency of PTAP duplication is considerably low in India as compared to the African countries. Using next generation sequencing, we could identify an absolute domination of the double-PTAP viral strains over the single-PTAP viral strains, in six of subjects containing a mixed infection, at several follow-up time points up to 3 years, in both the proviral DNA and plasma viral RNA compartments (unpublished data). Furthermore, using panels of infectious molecular clones that are genetically similar, we could demonstrate the domination of double-PTAP viral strain over the single-PTAP counterpart in the pairwise competition assay. Since the viral strains competing in the assay differ from each other only with respect to the presence of an additional PTAP motif, the replicative domination of the variant viral clone can be ascribed only to the presence of the additional PTAP motif (unpublished data). Additionally, using the proximity ligation assay, we could show an enhanced association between subtype C Gag p6 and Tsg101, an important member of the cell endosome sorting machinery. Thus, preliminary work from our laboratory is indicative of the significant replicative advantage conferred by PTAP motif duplication in subtype C. Importantly, unlike in subtype B, the prevalence of double-PTAP viral strains appears to be increasing in subtype C. The future studies must monitor the rate of prevalence of double-PTAP viral strains of subtype C. The biological significance of PTAP duplication whether such a variation offers replicative advantage to the variant viral strains has not been examined. The present study makes an important finding, offers an explanation for the replication fitness of the subtype C strains, and provides many important leads for future research.

## Abbreviations

ALIX: ALG-2 interacting protein
ART: Antiretroviral therapy
Env: Envelope
ESCRT: Endosomal sorting complexes required for transport
Gag: Group specific antigen
LTR: Long Terminal Repeat
Pol: Polymerase
RT: Reverse trancriptase
Tsg101: Tumor susceptibility gene 101
